# In Vitro Corrosion Behavior of Biodegradable Iron Foams with Polymeric Coating

**DOI:** 10.3390/ma13010184

**Published:** 2020-01-02

**Authors:** Radka Gorejová, Renáta Oriňaková, Zuzana Orságová Králová, Matej Baláž, Miriam Kupková, Monika Hrubovčáková, Lucia Haverová, Miroslav Džupon, Andrej Oriňak, František Kaľavský, Karol Kovaľ

**Affiliations:** 1 Department of Physical Chemistry, Faculty of Science, Pavol Jozef Šafárik University in Košice, Moyzesova 11, 041 54 Košice, Slovakia; 2 Institute of Geotechnics, Slovak Academy of Sciences, Watsonova 45, 040 01 Košice, Slovakia; 3 Institute of Materials Research, Slovak Academy of Sciences, Watsonova 47, 040 01 Košice, Slovakia

**Keywords:** iron foam, polyethyleneimine (PEI), biodegradation, powder metallurgy, coating

## Abstract

Research in the field of biodegradable metallic scaffolds has advanced during the last decades. Resorbable implants based on iron have become an attractive alternative to the temporary devices made of inert metals. Overcoming an insufficient corrosion rate of pure iron, though, still remains a problem. In our work, we have prepared iron foams and coated them with three different concentrations of polyethyleneimine (PEI) to increase their corrosion rates. Scanning electron microscopy (SEM) coupled with energy dispersive X-ray analysis (EDX), Fourier-transform infrared spectroscopy (FT-IR), and Raman spectroscopy were used for characterization of the polymer coating. The corrosion behavior of the powder-metallurgically prepared samples was evaluated electrochemically using an anodic polarization method. A 12 weeks long in vitro degradation study in Hanks’ solution at 37 °C was also performed. Surface morphology, corrosion behavior, and degradation rates of the open-cell foams were studied and discussed. The use of PEI coating led to an increase in the corrosion rates of the cellular material. The sample with the highest concentration of PEI film showed the most rapid corrosion in the environment of simulated body fluids.

## 1. Introduction

In recent years, the development of biodegradable orthopedical scaffolds has advanced significantly [1,2,3,4,5,6,7]. Resorbable materials are intended to serve as temporary support for damaged tissue. Compared to the standard medical devices typically made of stainless steel, cobalt-chromium, or titanium alloys [8], this new group of materials possess a particular advantage in the form of in vivo self-adsorbing capacity. Corrosion is therefore no longer seen as a problem, and appropriate biodegradable devices can be made by targeted designing and influencing of their degradation rates.

Iron-based biodegradable materials (Fe-BM) are considered a suitable alternative to permanent metallic implants [9,10,11,12,13,14,15]. They showed satisfactory cytocompatibility in previous studies and their mechanical properties could match those of natural bone [9,16,17]. Hydrogen evolution, too rapid degradation, or suppressed antibacterial performance, problems associated with the other most-studied biodegradable metal—magnesium, are not present in the case of iron [18,19]. However, the disadvantage of very slow degradation in physiological pH has to be overcome. There have been several reports studying the corrosion behavior of Fe-BM using different approaches to solve this issue. One of the most used methods to fasten the degradation is alloying with another element(s). Manganese, platinum, sulfur, carbon, palladium, etc. were tested in different ratios to the iron [10,15,20,21,22]. Even though these additions managed to accelerate iron degradation, mechanical properties or overall biocompatibility are often impaired. Degradation of BM depends on various factors and it is known that besides the composition of the specimen, the preparation method and its geometrical form plays also an important role [23]. The porous structure is beneficial for healthy vascularization and tissue ingrowth and is typically used in the field of orthopedic implants [24,25,26].

Another way to enhance corrosion, but also improve the biological performance of prepared material, is the usage of different coatings. Three groups of coating materials are usually used. The first group consists of inorganic ceramic coatings where hydroxyapatite (HAp) and other calcium phosphates (tricalcium phosphates (TCP), biphasic calcium phosphates (BCP)) have a leading position due to their similarity to the inorganic component of natural bone, osteoconductivity, and osseointegration properties [23,27,28]. Representatives of the second group are the metal–ceramic composites (calcium silicate-iron e.g., [29].)

Polymers are the third group of the coating materials for bioabsorbable metals. Poly-lactic-acid (PLA), poly-lactic-co-glycolic acid (PLGA), or polyethyleneglycol (PEG) are used to the highest extent [25,30,31]. It is known that the passivation layer of corrosion products can be formed on the surface of the specimen which retards further corrosion. Yusop et al. [31] found that pH in the proximity to the metal surface can be lowered by the polymer degradation and therefore the solubility of this passive layer is enhanced as long as the solubility of these corrosion products (mostly calcium or magnesium phosphates, iron hydroxides, etc.) is higher in the lower pH. This can lead to higher corrosion rates of the studied implants. Polymeric coating, though, can not only enhance the corrosion rate but also improve the biological performance of the scaffold. When PEG was used, the positive effect on the material biocompatibility was observed [32].

Polyethyleneimine (PEI) is an organic polymer soluble in water and ethanol with a high density of amino groups which can be protonated [33,34]. This polycation exists in linear or branched form and its properties depend on molecular weight and structure [35]. PEI has been studied for several decades [36] and found its place in various biological applications. It can be utilized as a drug carrier [35], in tumor imaging [37], or in gene transduction into mesenchymal stem cells (MSCs) [38]. The cytotoxic effect of the PEI relies upon the size, structure, and its ratio. Xia et al. [35] found, that by a careful selection of PEI size, it is possible to achieve minimal or no cytotoxicity. Yao et al. [38] confirmed that not only the size, but also the concentration of the coated layer, has an influence on the resulting cytotoxicity, which can be adjusted by careful choosing. Moreover, the polycationic character of PEI due to the amino groups’ protonation can interact with negatively charged bacteria [34]. Several studies confirmed improvement of the biocompatibility of PEI-coated materials [36,38]. In addition to this, the PEI structure provides possibilities to modify it with various polymers, create layers (e.g., PEG, chitosan), and furthermore, to load it with drugs that could possibly take a place in the bone healing process [34,38,39,40].

In our work, we have prepared foam-like scaffolds from the carbonyl iron powder (CIP) via the powder metallurgy process. Inspired by our previous work on Fe-PEG [30] material, we used polyethyleneimine as a coating material, which was deposited on the surface of the Fe sample using a cost-effective dip-coating method. The morphology of the sample’s surface was studied prior to coating and after depositing the PEI layer in three different concentrations. To determine the corrosion rate in a physiological environment, electrochemical potentiodynamic tests and in vitro immersion tests were carried out using Hanks’ solution to mimic body fluids. The composition and appearance of the corrosion products created after 4, 8, and 12 weeks of immersion were examined and the influence of the polymeric layer on corrosion of the iron scaffold was discussed.

## 2. Materials and Methods

### 2.1. Material Preparation

Porous iron samples were prepared from carbonyl iron powder (CIP) by BASF (type CC d50, 3.8–5.3 μm; 99.5% Fe, 0.05% C, 0.01% N, 0.18% O) by the impregnation of the polyurethane (PUR) foam (Filtren, TM 25133). The impregnating suspension consisted of 7 g of CIP iron powder, 6 mL of distilled water, and 0.2 g of gelatine (Sigma-Aldrich) dissolved at 60 °C for better adhesion of iron slurry to the PUR foam. Cylindrical (Ø 5 mm, h 15 mm) foams were impregnated for 24 h and thermally treated in a tube furnace (ANETA 1) at 450 °C for 1 h in N_2_ atmosphere (for PUR matrix elimination) and sintered at 1120 °C for 1 h in a reduction atmosphere (10% H_2_, 90% N_2_) to obtain the final structure. CIP pellets (Ø 10 mm, h 2 mm) used for Raman spectroscopy experiments were prepared by cold pressing iron powder at 600 MPa, subsequent sintering at 1120 °C for 1 h in a reduction atmosphere (10% H_2_, 90% N_2_), and coated as described below.

### 2.2. PEI Coating Preparation

Polyethyleneimine (Sigma-Aldrich; 50% (*w*/*v*) in H_2_O) film was achieved by a dip-coating process. Samples were ultrasonically cleaned in acetone and ethanol, in each for 10 min, and dipped into three different PEI solutions (5, 10, and 15 wt % corresponding to PEI1, PEI2, and PEI3, respectively) for 90 min and then dried at 37 °C for 12 h.

### 2.3. Microstructure and Surface Characterization

The microstructure of porous iron foams before and after 4, 8, and 12 weeks of corrosion was observed using an optical microscope (Olympus GX71, OLYMPUS Europa Holding GmbH, Hamburg, Germany). Samples were molded into the methyl-methacrylate resin (Dentacryl), hardened, and grinded.

Scanning electron microscopy (SEM, Jeol Ltd., Tokyo, Japan) and energy dispersive X-ray analysis (EDX) (JOEL JSM-7001F with INCA EDX analyzer, Oxford Instruments, Abingdon, Oxfordshire, UK) were used for surface morphology characterization.

The specific surface area (S_BET_) was determined by the low-temperature nitrogen adsorption method using a NOVA 1200e Surface Area and Pore Size Analyzer (Quantachrome Instruments, Hartley Wintney, UK). The values were calculated using Brunauer–Emmett–Teller (BET) theory.

The Fourier-transform infrared spectroscopy (FT-IR) spectra were recorded on the Tensor 29 infrared spectrometer (Bruker, Karlsruhe, Germany) using the attenuated total reflection (ATR) method.

The Raman spectra were recorded using a Renishaw inVia spectrophotometer (Renishaw UK Sales Ltd., Wotton-under-Edge, UK). All spectra were recorded through 4x-objective using a 532 nm laser from 100 to 4000 cm^−1^ at a 50% laser power. The samples were exposed to the laser for 10 s with 3 accumulations.

### 2.4. Electrochemical Corrosion Testing

The electrochemical measurements were conducted in Hanks’ solution (8 NaCl, 0.4 KCl, 0.14 CaCl_2_, 0.06 MgSO_4_.7H_2_O, 0.06 NaH_2_PO_4_.2H_2_O, 0.35 NaHCO_3_, 1.00 glucose, 0.60 KH_2_PO_4_, and 0.10 MgCl_2_.6H_2_O in g/L) with pH 7.4 ± 0.2 at 37 ± 1 °C using a potentiostat (Autolab PGSTAT 302N). A three-electrode system with Ag/AgCl/KCl (3 mol/L) as a reference electrode, platinum counter electrode, and iron sample as the working electrode were used. The potentiodynamic polarization tests were carried out from −1000 to −300 mV (vs. Ag/AgCl/KCl (3 mol/L)) at a scanning rate of 0.1 mV/s. The corrosion rate was determined using the Tafel extrapolation method and calculated from Equation (1), where *CR* is corrosion rate, *j_corr_* is corrosion current density (µA/cm^2^), *K* is a constant (3.27 × 10^−3^) determining output units of *CR*, *EW* is equivalent weight (27.92 g/eq for Fe), and *d* is the iron foam density (0.024 g/cm^3^ [41]).

(1) $$CR=\frac{j_{corr}K\;EW}{d}$$

### 2.5. Immersion Test

Before the static immersion test, all uncoated samples were ultrasonically cleaned in acetone and ethanol for 10 min, air-dried, and weighed. Static immersion tests were conducted for 12 weeks at 37 °C. Corrosion rates were calculated from Equation (2), where *m_f_* is sample weight after degradation (g), *m_i_* is sample weight at the beginning of the experiment (g), *K* is the constant (8.76 × 10^4^), *A* is the sample area (cm^2^), *t* is the exposure time (h), and *d* is the material density (g/cm^3^). Samples were immersed in 120 mL of Hanks’ solution and the uniform access of the corrosion medium to the whole sample surface was ensured.

(2) $$CR=\frac{\left(m_{i}-m_{f}\right)\;8.76\times 10^{-4}}{A\;t\;d}$$

pH of Hanks’ solution was measured, and total iron content was determined using atomic absorption spectroscopy on AAnalyst 100 after 4, 8, and 12 weeks of corrosion.

## 3. Results and Discussion

### 3.1. Material Characterization

#### 3.1.1. Morphology of the Sintered Iron Foam

**Figure 1:**
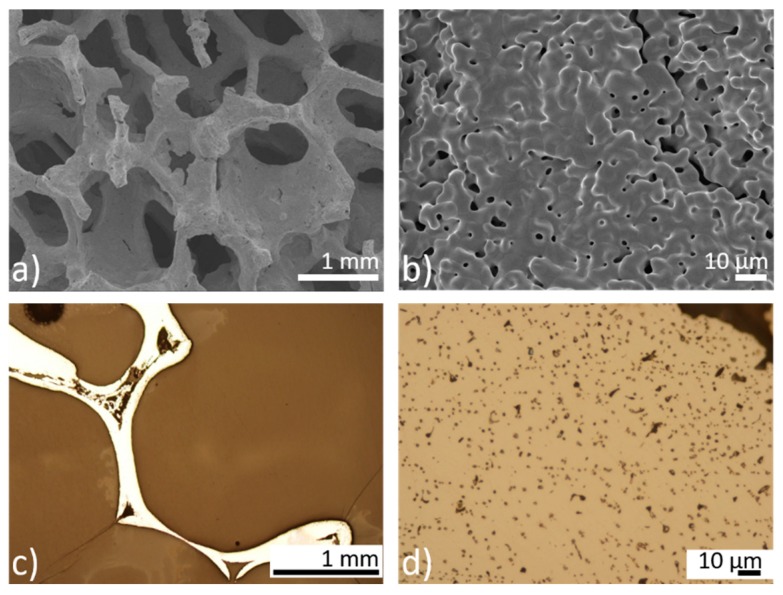
(**a**,**b**) The scanning electron microscopy (SEM) micrographs of the sintered iron scaffold; (**c**,**d**) metallographic cross-sections of the sintered iron scaffold. Comparison of the different porosities present in the material.

Cellular iron-based samples intended to serve as a potential orthopedic implant were prepared via the powder-metallurgical route. A little shrinkage of the specimens occurred after sintering when compared to the size of green compacts. Open porosity was well-preserved, which indicates a good material capacity for further tissue growth through the implant. Pores in the micrometer range (600 to 2000 μm) were present alongside smaller pores in the range of 0.5 to 6 μm, as shown in Figure 1. The surface of the sintered foams was humpy, as shown in Figure 1b, which can be attributed to the spherical character of the iron powder particles serving as raw material. Metallographic cross-sections of the pure iron foams, as shown in Figure 1c,d, confirmed these observations and showed a highly micro-porous structure.

Thinning of the cell walls occurred at their centers and the widest wall size was observed at the cell joints. Evaporation of gases after PUR foam elimination led to the creation of a third type of porosity, which was localized randomly, only in some regions of the samples, as shown in Figure 1c. This uncertain porosity should impair the mechanical properties of the specimen and should be considered and eliminated in the future fabrication process.

#### 3.1.2. Characterization of the Polymer Coating

**Figure 2:**
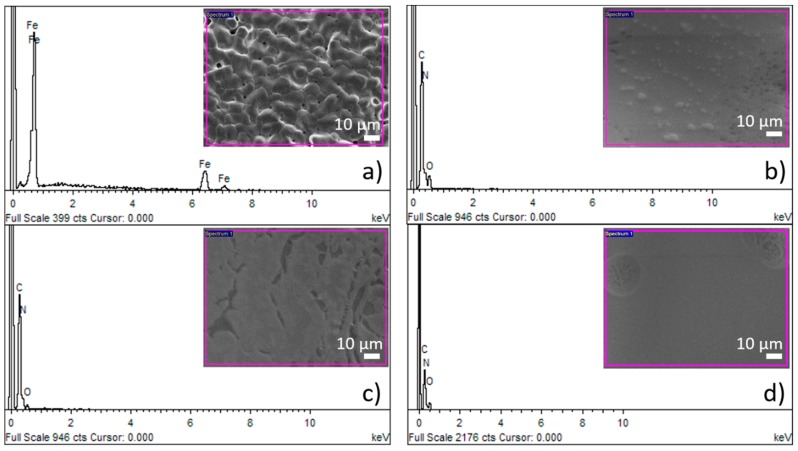
Chemical composition of the surface of (**a**) pure Fe; (**b**) Fe-PEI1; (**c**) Fe-PEI2; and (**d**) Fe-PEI3 samples studied by the energy dispersive X-ray analysis (EDX) method.

**Figure 3:**
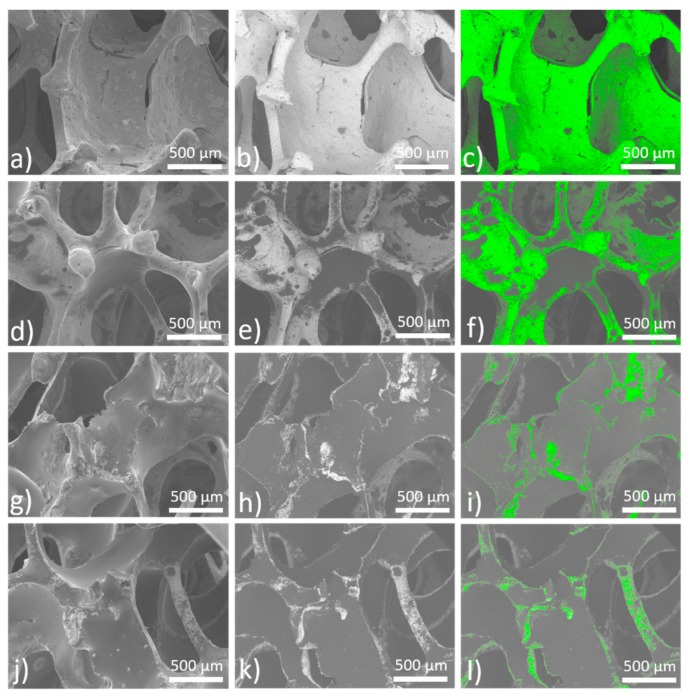
SEM micrographs of the prepared (**a**–**c**) Fe; (**d**–**f**) Fe-PEI1; (**g**–**i**) Fe-PEI2; and (**j**–**l**) Fe-PEI3 materials. Iron matrix is highlighted in green for better contrast in (**c**,**f**,**i**,**l**).

Sintered iron foams were ultrasonically cleaned and dip-coated with three different concentrations of PEI. The thin polymeric coating was observed after solvent evaporation. Ethanol (96 vol %) was selected as a solvent to achieve fast evaporation and to minimize the risk of material corrosion during the manufacturing processes. The presence of the polymeric layer was confirmed by the EDX method, where nitrogen, carbon, and oxygen were spotted for the coated samples while only iron was detected for the uncoated specimen, as shown in Figure 2. The average (from 10 measurements) weight of the resultant coating for different PEI concentrations and corresponding weight percentage is summarized in Table 1. While the PEI1 coating forms almost 2.0 wt % of the sample, it is 5.0 wt % for the PEI2 and 6.6 wt % for the PEI3 sample. The small difference between the weight of the PEI2 and PEI3 coating should be attributed to the higher saturation on the sample surface and depletion of the free space available for deposition. Similar space occupation by the polymeric layer for PEI2 and PEI3 can be seen in Figure 3i,l, while the uncoated areas are present when the sample is coated with only PEI1 (5 wt % of PEI) solution.

**Figure 4:**
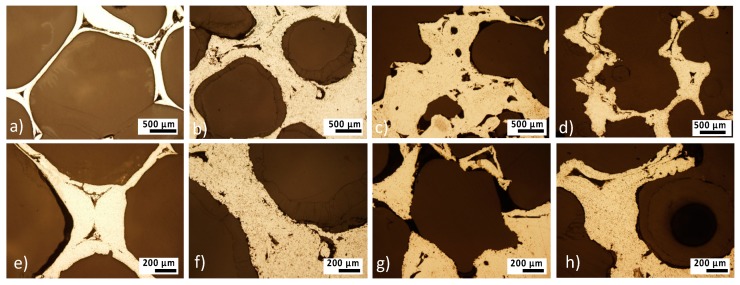
Metallographic cross-sections of the (**a**,**e**) Fe; (**b**,**f**) Fe-PEI1; (**c**,**g**) Fe-PEI2; and (**d**,**h**) Fe-PEI3 before corrosion.

Polymer distribution on the surface of iron matrix for the samples with different content of the polymer is depicted in Figure 3. SEM micrographs were taken in two different scanning modes (Secondary Electron Imaging (SEI) and Composition (COMPO)) for better evaluation of the polymer surface distribution. Difference between the heavy elements (e.g., Fe) and the light elements (e.g., C, O, N) is displayed as a color difference—the heavier is the element, the lighter is the color in which it is displayed. In Figure 3c,f,i,l, pure iron is graphically highlighted in green for better contrast. It can be seen that the coating on the Fe-PEI1 sample does not cover the entire surface and the polymer is mostly localized in the cell valleys, while the coverage of the wall edges is incomplete, as shown in Figure 3c. With the higher polymer concentration, coverage of the material increases, however, the edges of the walls still remain uncoated. Surface smoothing with increasing coverage of the iron substrate by the polymer layer led to the creation of the homogeneous surface. Polymer addition led to the micropores filling with coating material, as shown in Figure 4. The specific surface area (S_BET_) significantly decreased with the increasing polymer concentration, as shown in Table 2. This finding is similar to the results observed for the Fe-PEG material [30], where surface area increased after the first addition of polymer but decreased continuously with increasing polymer concentration. It can be seen that coating with PEI does not lead to the creation of islands of polymer and its use resulted in the smoothing of the coated surface. In the case of very porous substances with large specific surface areas, like activated carbon or fibrous silica, the addition of PEI also decreased the S_BET_ value [42,43]. The surface area plays an important role in the evaluation of the corrosion measurements and therefore should not be neglected in further analysis.

**Figure 5:**
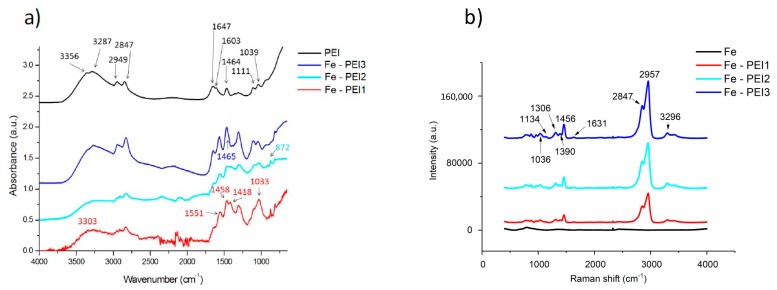
The spectra of pure iron and PEI coated scaffolds recorded using (**a**) FT-IR and (**b**) Raman spectroscopy.

The polymeric layer was confirmed and analyzed by different methods. FT-IR and Raman spectra were recorded before the corrosion of material to study the PEI layer and its interaction with the Fe matrix, as shown in Figure 5.

The vibrations of the functional groups of PEI can be found at the following wavenumber in the spectrum of pure PEI: the stretching vibrations of the –NH_2_ group at 3356 and 3287 cm^−1^, the asymmetric and symmetric stretching vibrations of –CH_2_ group at 2949 and 2847 cm^−1^, the bending vibration of the –NH_2_ group at 1603 cm^−1^, the in-plane bending vibration of the –CH_2_ group at 1464 cm^−1^, and the stretching vibration of the C–N group at 1111 cm^−1^. These positions are in accordance with recent literature [44,45,46]. After the interaction of PEI with Fe, the spectrum has significantly changed, as the majority of the peaks were shifted and also their intensity changed, as shown in Figure 5a. Moreover, there are differences among the Fe-PEI samples. Basically, for the samples with the low PEI content, the intensity of the peaks corresponding to the vibrations of the –NH_2_ group has significantly decreased in the case of these samples, meaning that this group can be mainly responsible for the interaction with iron. Furthermore, the new peak located at 872 cm^−1^ was evidenced. It is possible that the most effective interaction between Fe and the polymer is achieved when a smaller amount of PEI is used.

Figure 5b depicts the Raman spectrum of the PEI in different concentrations deposited on the surface of the CIP pellets. The bands at 1456 cm^−1^ and bands at 1306, 1134, and 1036 cm^−1^ correspond to the methylene –CH_2_ group (wagging and twisting motions) and could be also found in the spectra of the pure polyethylene and ethylenediamine [47]. The 1456 and 1306 cm^−1^ bands are also present in the spectrum of the pure 50 wt % PEI solution, which was observed in [47]. Bands at the 1631 cm^−1^ correspond to the amino group (–NH_2_). Intensive bands corresponding to the C–H bond are present at 2700 to 3100 cm^−1^, which is in accordance with the literature [48]. Different conformational changes could appear during the polymer adsorption to the surface. Symmetric and asymmetric valence vibrations are slightly shifted in the spectrum due to the amino groups in the PEI structure. In the pure 50 wt % PEI spectrum are these bands at 2956 and 2866 cm^−1^, but due to the adsorption processes to the metallic surface, these could be shifted (from 2866 cm^−1^ to 2847 cm^−1^ in this case). Chaufer [49] studied PEI adsorption onto Zr and assumed that the Lewis acid–base bonding occurs between the amino groups and the metal, which was also reported in the case of silver [47]. However, the phenomenon was observed when the –NH_2_ group band (1600 cm^−1^) was shifted to the lower values. We have observed shift to the higher values (1631 cm^−1^), therefore this type of interaction probably could not be applied to our system. Despite this fact, the analysis of the FT-IR spectra of coated foams discussed earlier in this work confirmed the interaction between the amino group and the metal surface.

### 3.2. Degradation Study

#### 3.2.1. Potentiodynamic Polarization Tests

**Figure 6:**
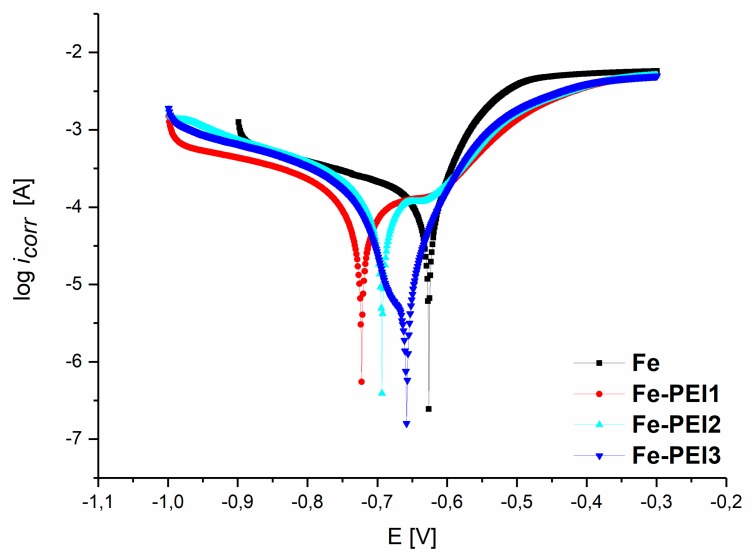
Polarization curves of the iron-based foams with or without PEI coating obtained in the Hanks’ solution at 37 ± 1 °C.

For the evaluation of the degradation rates of coated and uncoated samples, a potentiodynamic polarization test was performed. Corrosion current density (*j_corr_*), corrosion potential (*E_corr_*), and the polarization resistance are summarized in Table 3. Potentiodynamic curves obtained during the measurement in the Hanks’ solution at 37 ± 1 °C from −1000 to −300 mV are shown in Figure 6.

The coating of the iron foams with the PEI has resulted in the shift of the corrosion potential to the more negative values obtained for all three different concentrations of polymer. The lowest value of *E_corr_* was observed for the Fe-PEI1, followed by the Fe-PEI2, Fe-PEI3, and the pure iron, which exhibited the most positive value (−627.0 mV). Reported corrosion potentials of the pure iron observed by the anodic polarization method were −860.7 mV [41], −484.0 mV [50] or −510.0 mV [51]. For example, in [52], the authors studied electrochemical degradation of the pure iron bars and phosphated iron bars in Hanks’ solution and observed the corrosion potential of pure iron to be −670 mV, which is similar to that observed in this study. It can be seen, from the different results for the same composition of the sample (Fe), that the corrosion rate is dependent also on the preparation method and sample geometry. The foam-like structure of the samples prepared by the powder metallurgy method resulted in the shift of the electrochemical corrosion potential to more negative values when compared to that of standard pure iron.

The highest corrosion current density was observed for the Fe-PEI3 sample, indicating the highest ability to corrode. Corrosion rates (*CR*) calculated from Equation (1) confirmed that the highest corrosion rate from all the samples was the Fe-PEI3 sample, even though its polarization resistance (*PR*) was the highest when compared to other samples. The determining parameter for such a behavior is the material surface area, as shown in Table 2. The specific surface area of the pure iron is, due to its inhomogeneity, almost 30 times higher when compared to the Fe-PEI3. This fact emphasizes the need to know the real surface areas during the evaluation of degradation behavior of polymer-coated samples with the tendency to lower S_BET_ values. Porous iron coated with PLGA [31] reached the corrosion rate of 0.420 mm y^−1^, which is similar to the results of Fe-PEI3 material (0.590 mm y^−1^). An important difference can be seen in the corrosion rates of pure iron, which is 5 times lower than that reported in [53]. This fact can be also attributed to the different surface areas of the pure iron sample affected by the preparation method and to the highly-porous structure. Similar values of *CR* (0.04 ± 0.01) were observed in [16] for 3D-printed Fe-Mn samples, while in [51], the authors reported the *i_corr_* of pure Fe to be 1.68 × 10^−5^ A, which emphasizes even more the influence of the preparation method on the degradation behavior of biodegradable materials.

#### 3.2.2. Static Degradation Tests

**Figure 7:**
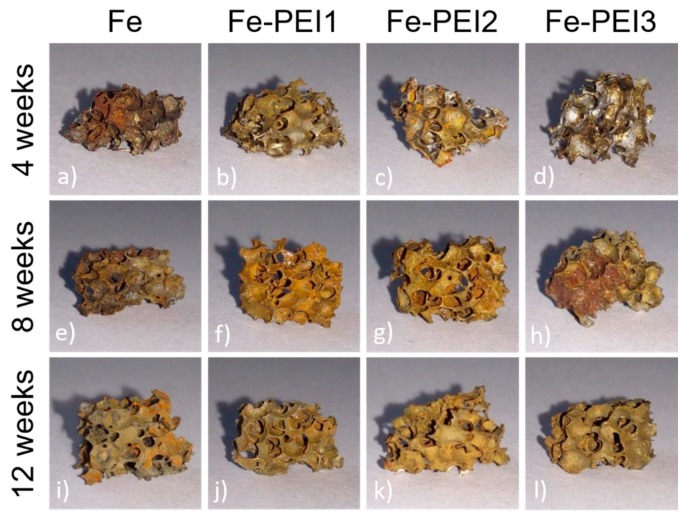
Cellular scaffold made of (**a**,**e**,**i**) pure Fe; (**b**,**f**,**j**) Fe-PEI1; (**c**,**g**,**k**) Fe-PEI2; and (**d**,**h**,**l**) Fe-PEI3 after a static immersion test in Hanks’ solution at 37 °C.

**Figure 8:**
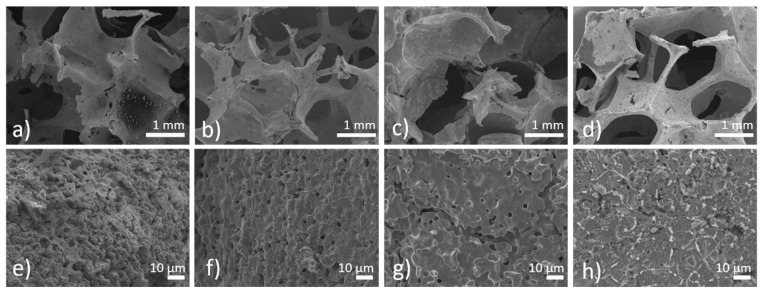
The SEM micrographs of the **(a**,**e**) Fe; (**b**,**f**) Fe-PEI1; (**c**,**g**) Fe-PEI2; and (**d**,**h**) Fe-PEI3 samples after 4 weeks of corrosion in the Hanks´ solution at 37 °C.

**Figure 9:**
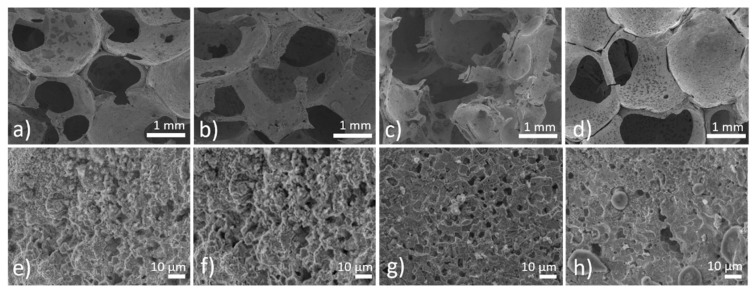
The SEM micrographs of the (**a**,**e**) Fe; (**b**,**f**) Fe-PEI1; (**c**,**g**) Fe-PEI2; and (**d**,**h**) Fe-PEI3 samples after 12 weeks of corrosion in the Hanks´ solution at 37 °C.

**Figure 10:**
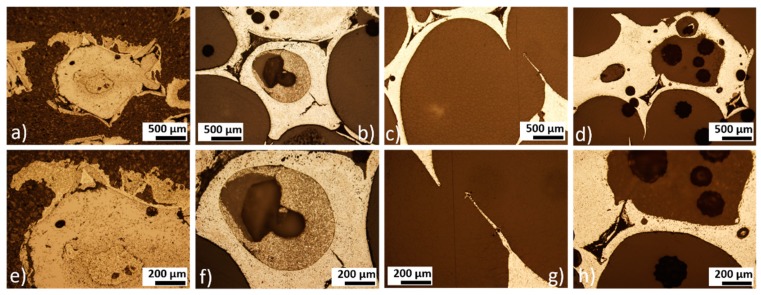
Metallographic cross-sections of the (**a**,**e**) pure iron; (**b**,**f**) Fe-PEI1; (**c**,**g**) Fe-PEI2; and (**d**,**h**) Fe-PEI3 after 12 weeks of corrosion.

Static immersion tests provide complex information about the degradation processes of metallic samples and also about the changes in the corrosion medium. The most important advantage of this type of corrosion testing is its ability to simulate real-body conditions in a more authentic way than dynamic electrochemical tests. Pictures of iron and PEI-coated iron foams after degradation tests are shown in Figure 7. After 8 weeks of corrosion, all samples were completely covered with corrosion products in brown, red, and orange color forming the rust. With the prolonged time of immersion, surface roughness and inhomogeneity increased for all samples, as shown in Figure 8, Figure 9 and Figure 10. Uniform corrosion, as shown in Figure 8a,b, was observed in the initial stage of degradation whereas pitting corrosion, as shown in Figure 9f,g, typical for the environment with high concentration of chloride ions [54], occurred with prolonged time of immersion. In Figure 10 are presented cross-sections of the Fe and Fe-PEI samples after 12 weeks of corrosion. The main difference, compared to the un-corroded material shown in Figure 4, could be observed in the thinning of the cell walls accompanied by cracking at the narrowest points, as shown in Figure 10c,d, which can lead to the worsening of the mechanical properties of such material.

Corrosion rates of the coated and uncoated iron-based foams were calculated from the weight loss values recorded after 4, 8, and 12 weeks. The slowest corrosion in the Hanks’ solution at 37 °C was observed for the pure iron, followed by the Fe-PEI1, Fe-PEI2, and Fe-PEI3, which is in accordance with the results obtained for Fe and Fe-PEI3 during the potentiodynamic tests. *CR* at different stages of the immersion experiment is listed in Table 4. Yusop et al. [31] have studied PLGA-coated iron and found the corrosion rate to be 0.76 mm y^−1^, which is comparable to that of Fe-PEI3 determined in this study, as shown in Table 4. The degradation rate of pure iron did not change significantly during the testing period, whereas the *CRs* of Fe-PEI1 and Fe-PEI2 decreased with prolonged time. The Fe-PEI3 sample corroded fastest for the second month and its degradation slowed down during the third month due to the formation of a passivation layer of corrosion products. The thickest polymer layer deposited on the Fe-PEI3 sample could, therefore, serve as a corrosion barrier in the initial stage of the degradation process.

**Figure 11:**
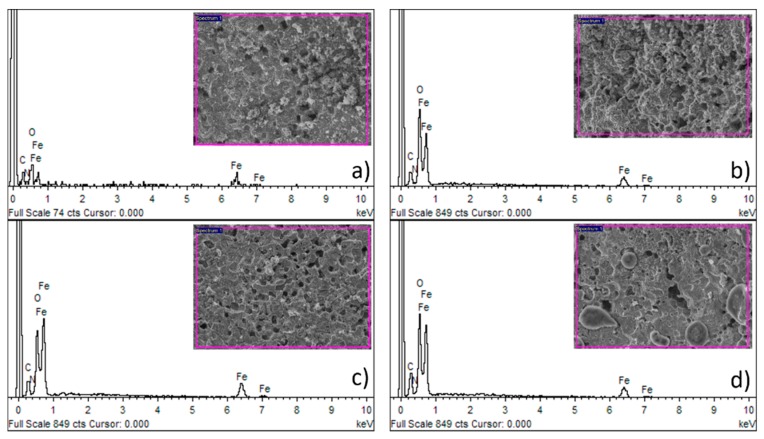
The chemical composition of the surface of (**a**) pure Fe; (**b**) Fe-PEI1; (**c**) Fe-PEI2; and (**d**) Fe-PEI3 after 12 weeks of corrosion in simulated body fluids studied by the EDX method.

**Figure 12:**
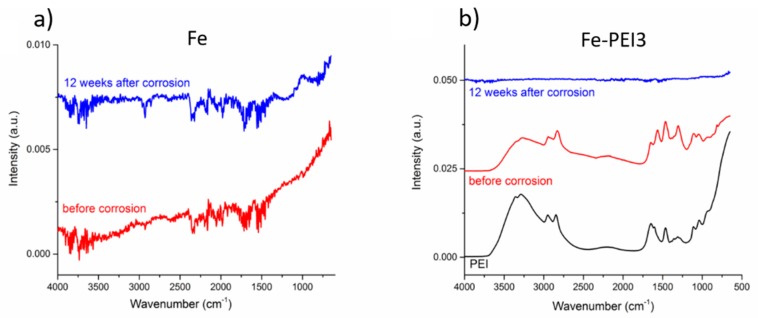
FT-IR spectra of (**a**) pure Fe; (**b**) Fe-PEI3 after 12 weeks of corrosion in Hanks’ solution. Spectrum of pure PEI is added for better comparison.

The composition of the iron-based sample surface after 12 weeks of degradation is shown in Figure 11. All the samples were completely covered with the corrosion products comprising mostly of iron hydroxides. No evidence of nitrogen was observed, assuming total degradation of the PEI coating after three months, which was also confirmed by the FT-IR analysis, as shown in Figure 12.

**Figure 13:**
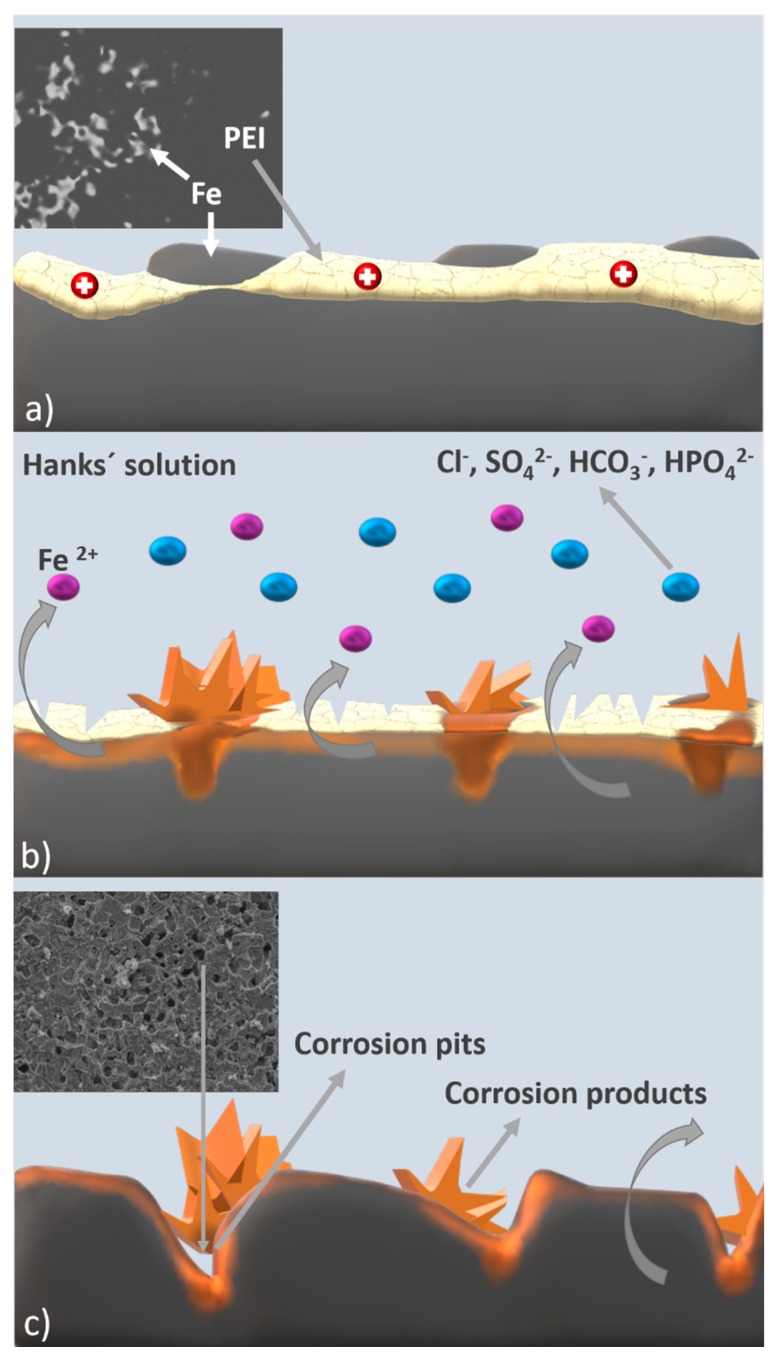
Schematic representation of corrosion processes ongoing on the surface of the Fe-PEI material. (**a**) Polymer-coated sample on-air; (**b**) polymer-coated sample after immersion into Hanks’ solution—PEI layer disruption; (**c**) formation of corrosion pits after 12 weeks of biodegradation.

The influence of the corrosion environment on the Fe and Fe-PEI was investigated by FT-IR, as shown in Figure 13a,b, respectively. In Figure 13b, the spectrum of PEI is also included for comparison. The individual intensities of the spectra were adjusted in order to create a comparable figure, namely, the intensities of the polymer and Fe-PEI samples were decreased 10 and 5 times, respectively. The spectra of iron, as shown in Figure 13a, before and after corrosion are almost identical; the differences stay within the measurement error. Neither of the samples exhibit a significant band in FT-IR. The difference between the spectrum of the pure PEI and Fe-PEI specimen was already described earlier. After 12 weeks of corrosion, no bands can be observed, thus the organic layer was completely decomposed. Reactions ongoing during iron degradation in Hanks’ solution were already described [23].

Yusop et al. [31] have suggested accelerated corrosion as a consequence of lowering of pH induced by the polymer hydrolysis. The pH of the Hanks’ solution was measured during the immersion study and results are listed in Table 5. After 4 weeks, pH of the corrosion medium was slightly shifted to the higher values. In the case of pure iron and the Fe-PEI3 sample, the constant rise of pH was observed with resultant values of 8.06 and 8.73, respectively. pH values of all of the tested samples were higher at the end of the immersion study and exceeded the value of pH = 8. More basic pH can be attributed to the creation of the corrosion products (e.g., Fe(OH)_2_, Fe(OH)_3_, FeCl_2_OH). Fe-PEI1 and Fe-PEI2 degradation resulted in the lowering of the pH after 8 weeks of the immersion. It is possible that in the case of higher PEI concentration (PEI3), the degradation of the polymeric layer is more rapid in the initial stage (before the fourth week). It increases solubility of the layer of corrosion products, which results in enhanced corrosion, and therefore in the higher content of corrosion products in the Hanks’ solution responsible for basic pH. As long as PEI can act as a buffering agent, residual acidity created at the beginning of the degradation process can be compensated by the protonation of PEI functional amino groups.

Degradation of the polymer caused creation of the localized cracks and pits which served as an entrance for corrosive medium into the depth of the sample, as shown in Figure 13. This can explain the corrosion rate enhancement of coated samples and presence of the pitting corrosion. Moreover, chelation processes between polymer and metal ions [55] could contribute to the higher content of the iron in the solution. Atomic absorption spectroscopy was used to determine the total concentration of the iron (mg mL^−1^) in the medium, as shown in Table 6. All the observed values increased with prolonged time of immersion, with the highest amount of 0.0337 mg mL^−1^ for Fe-PEI3. Information about the iron concentration released into solution is important for further determination of material cytotoxicity.

Zhang [51] reported that iron concentration lower than 0.075 mg mL^−1^ is safe for cells, therefore it can be assumed that biodegradable foams studied in this paper should not possess cytotoxicity based on the amount of released metal ions into the body. Cytotoxicity and hemocompatibility studies of the material are therefore necessary for its further evaluation.

## 4. Conclusions

Iron-based foams with open-cell porosity were prepared and analyzed. The deposition of the polymeric (PEI) layer on the surface of the samples led to the changes in their morphology. A significant decrease in the surface area was observed after the application of coating (from 1.19 mg m^−2^ for pure iron to 0.04 mg m^−2^ for Fe-PEI3). Moreover, desirable corrosion rate enhancement mediated through the polymer cracking and corrosion medium penetration enabling took place in the case of polymer-coated samples.

Based on the results reported in this study, coating with polymers can lead to changes in the corrosion rates of metallic samples. Variations in their concentrations seem to be an appropriate way to design devices with desired degradation behavior. PEI is a flexible polymer that can be further functionalized, which makes a room for its future modification. Loading PEI with drugs that could help in bone treatment processes should be an interesting challenge for future research.

## Figures and Tables

**Table 1:** Average weight (mg) and content (wt %) of polyethyleneimine (PEI) coating deposited on the surface of the iron foams.

	Fe-PEI1	Fe-PEI2	Fe-PEI3
Average PEI weight (mg)	15.9	41.4	53.6
Average PEI content (wt %)	1.9	5.0	6.6

**Table 2:** Specific surface area (S_BET_) of the PEI coated (Fe-PEI) and the uncoated (pure Fe) foams.

S_BET_ (g m ^−2^)			
**Fe**	**Fe-PEI1**	**Fe-PEI2**	**Fe-PEI3**
1.19	0.92	0.61	0.04

**Table 3:** Electrochemical parameters of the Fe and Fe-PEI samples obtained from the Tafel analysis of polarization curves measured at 37 ± 1 °C in the Hanks’ solution. Corrosion current (***i_corr_***), corrosion current density (*j_corr_*), corrosion potential (*E_corr_*), polarization resistance (*PR*), corrosion rate (*CR*).

Sample	*E_corr_* (V)	*i_corr_* (A)	*j_corr_* (μA cm^−2^)	*PR* (Ω cm^−2^)	*CR* (mm y^−1^)
Fe	−0.627	11.91 × 10^−5^	1.18 × 10^−2^	0.017	0.045
Fe-PEI1	−0.722	37.11 × 10^−5^	4.52 × 10^−2^	0.041	0.172
Fe-PEI2	−0.687	102.95 × 10^−5^	3.10 × 10^−2^	0.039	0.118
Fe-PEI3	−0.658	5.39 × 10^−5^	15.49 × 10^−2^	4.460	0.590

**Table 4:** Corrosion rates of Fe, Fe-PEI1, Fe-PEI2, and Fe-PEI3 calculated from the weight-loss experiments in Hanks’ solution at 37 °C for 12 weeks.

*CR* [mm y ^−1^]			
Week of Immersion	4	8	12
Fe	0.004 ± 0.0015	0.005 ± 0.0030	0.005 ± 0.0034
Fe-PEI1	0.024 ± 0.0052	0.006 ± 0.0008	0.015 ± 0.0047
Fe-PEI2	0.148 ± 0.0420	0.037 ± 0.0113	0.021 ± 0.0209
Fe-PEI3	0.697 ± 0.0398	1.547 ± 0.0793	0.199 ± 0.0109

**Table 5:** pH of Hanks’ solution after 4 to 12 weeks long immersion of iron-based samples coated with PEI and pure iron foams.

pH ± 0.2				
Week of Immersion	0	4	8	12
Fe	7.40	7.46	7.75	8.06
Fe-PEI1	7.40	7.43	7.06	8.08
Fe-PEI2	7.40	7.69	7.53	8.23
Fe-PEI3	7.40	7.48	8.25	8.73

**Table 6:** Total iron concentration observed in the Hanks’ solution after 4, 8, and 12 weeks of immersion of cellular scaffolds with polymer (PEI) coating.

Total Iron Concentration (mg mL ^−1^)			
Week of Immersion	4	8	12
Fe	0.0243	0.0265	0.0316
Fe-PEI1	0.0151	0.0278	0.0284
Fe-PEI2	0.0184	0.0224	0.0251
Fe-PEI3	0.0139	0.0283	0.0337
